# How to remove the grey area between ventilator-associated pneumonia and ventilator-associated tracheobronchitis?

**DOI:** 10.1186/s13054-017-1754-6

**Published:** 2017-07-08

**Authors:** Yuetian Yu, Cheng Zhu, Chunyan Liu, Yuan Gao

**Affiliations:** 1 Department of Critical Care Medicine, Ren Ji Hospital, School of Medicine, Shanghai Jiao Tong University, 145, Middle Shangdong Road, Shanghai, 200001 China; 2 Department of Emergency Medicine, Rui Jin Hospital, School of Medicine, Shanghai Jiao Tong University, Shanghai, 200025 China; 3 Department of Emergency Medicine, Min Hang Hospital, School of Medicine, Fu Dan University, Shanghai, China

We read with great interest the study performed by Paula Ramirez and colleagues [1]. The study included 71 patients with ventilator-associated pneumonia (VAP) and the authors coined a new term called “gradual VAP”. The result of this study indicated that an early antibiotic treatment for patients with gradual VAP was associated with an increased rate of early clinical response. Based on the results of previous studies and our observational data, we would like to make some comments.

There is still a grey area between ventilator-associated tracheobronchitis (VAT) and VAP in clinical practice, resulting in a situation where ventilator-associated event (VAE) and infection-related ventilator-associated complication (IVAC) are proposed as alternative terms for epidemiologic purposes [2, 3]. The nature of subjectivity and variability of chest radiograph interpretation makes chest imaging ill-suited for the definition of IVAC. However, it is challenging for the clinician to make a diagnosis of pneumonia without chest imaging, leading to the fact that the IVAC definition is mainly used in surveillance. Thus, it is a challenge to determine the timing of the initiation of antibiotic treatment.

Gradual VAP is a novel concept that might be a better bridge to link VAT and VAP than IVAC. Based on the 2016 clinical practice guidelines issued by the Infectious Diseases Society of America [4], 82 patients were diagnosed as VAP and enrolled in our prospective observational study from July 2016 to March 2017 in three teaching hospitals (Ren Ji, Rui Jin, and Minhang hospitals; 157 intensive care unit (ICU) beds in total). Among the VAP patients, 42 had gradual VAP, 34 had IVAC, and the remaining 6 patients had neither. The baseline characteristics were not significantly different between patients with gradual VAP and IVAC. Characteristics at the time of VAP diagnosis and outcome of VAP patients are compared in Table 1 [5].

**Table 1:** Characteristics at time of VAP diagnosis and outcome of VAP patients

	Gradual VAP (*n* = 42)	IVAC (*n* = 34)	*P* value
At the time of VAP diagnosis			
MV duration before VAP, days	9.32 ± 2.35	9.02 ± 3.51	0.658
Early VAP	6 (14.29)	0 (0)	0.030
SOFA score	7.32 ± 2.31	8.01 ± 3.19	0.278
mCPIS score	7.19 ± 1.92	7.33 ± 2.03	0.759
PaO_2_/FiO_2_, mmHg	160.32 ± 25.17	151.12 ± 19.19	0.083
Temperature, °C	38.19 ± 1.08	38.23 ± 0.92	0.864
WBC, ×10^9^/L	13.42 ± 3.28	12.12 ± 3.06	0.096
Outcome			
Empiric treatment failure (72 h)	5 (11.90)	11 (32.35)	0.046
ICU stay, days	18.32 ± 5.19	20.78 ± 4.73	0.029
Hospital stay, days	28.17 ± 6.32	31.92 ± 8.14	0.036
Days of MV	15.23 ± 5.42	18.39 ± 7.14	0.031
Total antibiotics expenditures,^USD^	4058.34 ± 273.56	5425.89 ± 385.74	<0.001

Results are expressed as mean ± SD for continuous variables and number (%) for categorical variables

*USD* United States dollar

*ICU* intensive care unit, *IVAC* infection-related ventilator-associated complication, *mCPIS* modified Clinical Pulmonary Infection Score, *MV* mechanical ventilation, *PaO*_*2*_ */FiO*_*2*_ ratio of partial pressure of arterial oxygen to fraction of inspired oxygen, *SOFA* Sepsis-related Organ Failure Assessment, *VAP* ventilator-associated pneumonia, *WBC* white blood cell

The definition of IVAC includes “a new antimicrobial agent is started, and is continued for ≥4 calendar days”, which is the main difference to gradual VAP [3]. Furthermore, the definition criteria do not include changes in chest imaging. Our brief report indicated that starting empiric antibiotic treatment at the period of gradual VAP was associated with short ICU and hospital length of stay. The rate of antibiotic treatment failure and cost were also lower than IVAC. In conclusion, gradual VAP still needs to be well-defined so that it can be an intermediate form of ventilator-associated infection linking VAT and VAP.

## Abbreviations

IVAC: Infection-related ventilator-associated complication
VAE: Ventilator-associated event
VAP: Ventilator-associated pneumonia
VAT: Ventilator-associated tracheobronchitis
